# Multi-ethnic evaluation of fully automatic planning assist system for cardiac magnetic resonance imaging

**DOI:** 10.1186/1532-429X-16-S1-P76

**Published:** 2014-01-16

**Authors:** Shigehide Kuhara, Shuhei Nitta, Taichiro Shiodera, Yukinobu Sakata, Tomoyuki Takeguchi, Kenichi Yokoyama, Reiko Ishimura, Toshiya Kariyasu, Masamichi Imai, Toshiaki Nitatori, Timothy Albert

**Affiliations:** 1 Toshiba Medical Systems Corporation, Otawara-shi, Japan; 2 Corporate Research & Development Center, Toshiba Corporation, Kawasaki-shi, Japan; 3 Department of Radiology, Kyorin University, Faculty of Medicine, Mitaka-shi, Japan; 4 Advanced Diagnostic Imaging Center, Salinas Valley Memorial Hospital, Monterey, California, USA

## Background

We have been developing an automatic planning assist system for coach adjustment, local shimming scan plan, axial multi slice scan plan for slice-alignment, and whole-heart MR imaging plan (motion probes and an axial slab of main scan) [1,2]. However, the previous reposts of the system evaluated only Japanese patient's and healthy volunteer's datasets. The purpose of this study is to evaluate the accuracy for multi-ethnic datasets based on the inter-observer error in manual annotation.

## Methods

An ECG-non-gated 3D fast field echo (FFE) single volume covering the entire chest area was acquired using a 1.5-T MRI scanner (Excelart VantageTM powered by Atlas, Toshiba Medical Systems) during a single breath-hold with TR/TE = 3.7/1.3, FOV = 500 × 350 × 350 mm 3 (coronal slab), and readout/phase/slice encode steps = 256/64/35 in an acquisition time of approximately 9 seconds. The proposed system employs an accurate and quick registration technique, the input volume is registered to a prepared model volume which is a well-rounded physical-size. By the registration, the cardiac region and the top of the right hemidiaphragm position of the input volume were detected because the prepared model volume has correct information of the region and the position. The detected region was used for settings of coach adjustment, local shimming scan plan, multi slice scan plan and whole-heart MR imaging scan plan (an axial slab of main scan). The detedted position was used for motion probes setting in whole-heart MR imaging. In order to evaluate detection results, the Euclidean distance errors of the six sides of the circumscribed cuboids of the cardiac region and the position of the top of the right hemidiaphragm between detection results and manual annotations were measured. In addition, the inter-observer errors between two manual annotations were measured.

## Results

**Figure 1:**
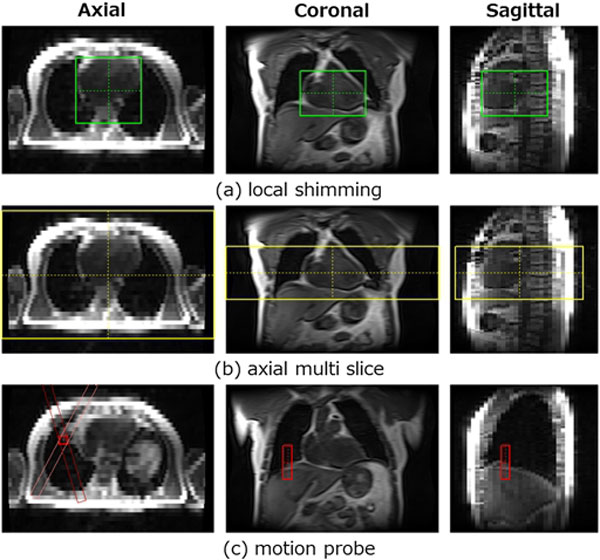
**Example of the detection results for American clinical data**.

**Figure 2:**
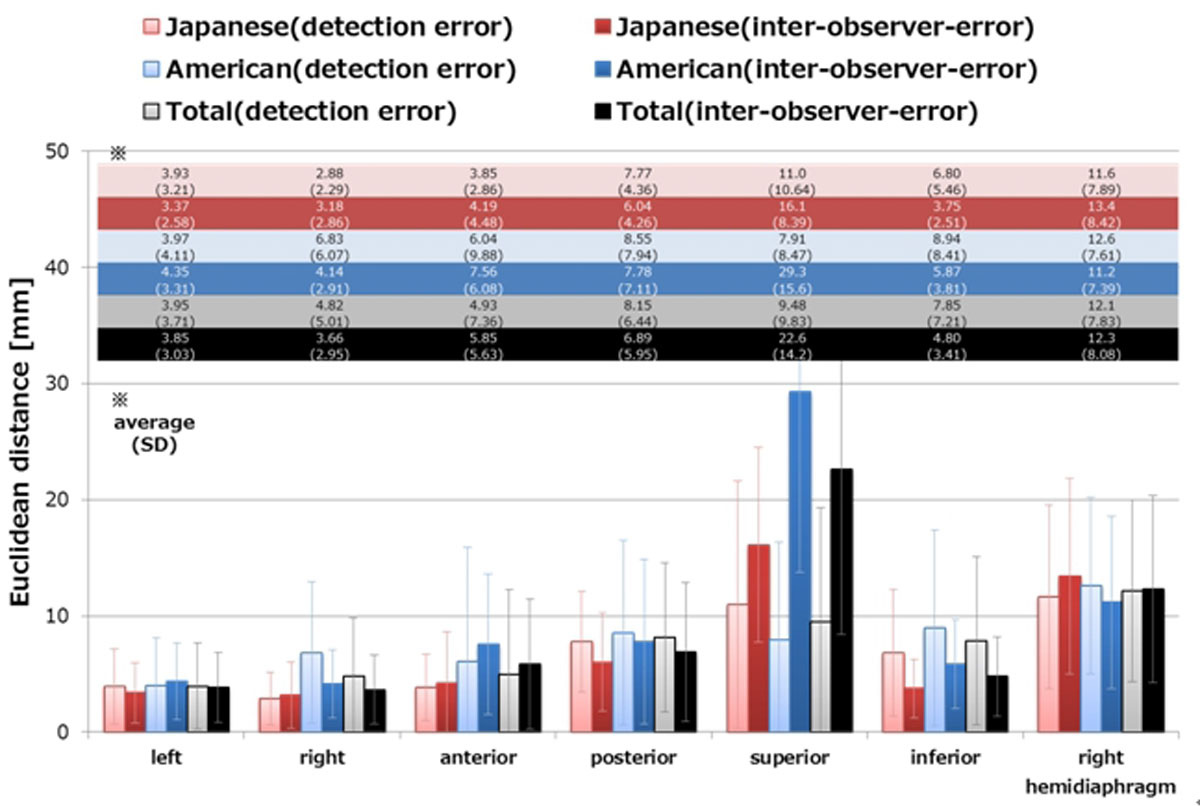
**Average and standard deviation (SD) of the Euclid distance errors**.

The proposed method was performed in 32 datasets from 32 Japanese patients, and 31 datasets from 31 American patients and there were no undetectable subjects. An example of the detection results and the average and standard deviation of the distance and inter-observer errors are shown in Figure 1 and Figure 2, respectively. The processing time was about 1.6 seconds on a 2.5 GHz CPU.

## Conclusions

The proposed system is useful for a variety of ethnic groups.

## Funding

No funding was received for this research.

